# Sustained inactivation of the Polycomb PRC1 complex induces DNA repair defects and genomic instability in epigenetic tumors

**DOI:** 10.1007/s00418-024-02302-z

**Published:** 2024-06-18

**Authors:** Chetan C. Rawal, Vincent Loubiere, Nadejda L. Butova, Juliette Gracia, Victoria Parreno, Chiara Merigliano, Anne-Marie Martinez, Giacomo Cavalli, Irene Chiolo

**Affiliations:** 1 Department of Molecular and Computational Biology, University of Southern California, 1050 Childs Way, Los Angeles, CA 90089 USA; 2 Institute of Human Genetics, CNRS, University of Montpellier, Montpellier, France; 3 Research Institute of Molecular Pathology, Vienna BioCenter, Vienna, Austria

**Keywords:** Epigenetically initiated cancers, Polycomb complex, Double-strand break repair, Genomic instability

## Abstract

Cancer initiation and progression are typically associated with the accumulation of driver mutations and genomic instability. However, recent studies demonstrated that cancer can also be driven purely by epigenetic alterations, without driver mutations. Specifically, a 24-h transient downregulation of *polyhomeotic* (*ph*-KD), a core component of the Polycomb complex PRC1, is sufficient to induce epigenetically initiated cancers (EICs) in *Drosophila*, which are proficient in DNA repair and characterized by a stable genome. Whether genomic instability eventually occurs when PRC1 downregulation is performed for extended periods of time remains unclear. Here, we show that prolonged depletion of PH, which mimics cancer initiating events, results in broad dysregulation of DNA replication and repair genes, along with the accumulation of DNA breaks, defective repair, and widespread genomic instability in the cancer tissue. A broad misregulation of H2AK118 ubiquitylation and to a lesser extent of H3K27 trimethylation also occurs and might contribute to these phenotypes. Together, this study supports a model where DNA repair and replication defects accumulate during the tumorigenic transformation epigenetically induced by PRC1 loss, resulting in genomic instability and cancer progression.

**Supplementary Information:**

The online version contains supplementary material available at 10.1007/s00418-024-02302-z.

## Introduction

The tumorigenic process is typically associated with DNA damage defects and genomic instability (Hopkins et al. 2022). However, recent studies established that cancer can also be induced purely by epigenetic changes initiated by the transient loss of the transcriptionally repressive Polycomb PRC1 complex (Parreno et al. 2024). Polycomb Group (PcG) proteins are grouped in two main classes of complexes called Polycomb Repressive Complex 1 and 2 (PRC1 and PRC2) (Levine et al. 2002; Kassis et al. 2017; Schuettengruber et al. 2017). *Drosophila* PRC1, which is composed of PH, PC, PSC, and SCE subunits, is primarily responsible for H2AK118 ubiquitylation (H2AK118ub, corresponding to H2AK119ub in mammals) (Barbour et al. 2020; Parreno et al. 2022), whereas PRC2 mediates H3K27 trimethylation (H3K27me3) (Holoch and Margueron 2017). PRC1 and PRC2 are also highly interdependent, given that PRC1 binds to H3K27me3 while PRC2 associates with H2AK118ub (Blackledge et al. 2014; Cooper et al. 2016, Kasinath et al. 2021). This enables cooperative binding of the two complexes to the same sites and codependency in the establishment of the respective marks on chromatin (Cooper et al. 2016; Barbour et al. 2020, Tamburri et al. 2020). While PRC1 and PRC2 form several redundant subcomplexes in mammalian cells (Parreno et al. 2022, Potter et al. 2023), *Drosophila* Polycomb complexes comprise a reduced number of paralogous and accessory subunits, facilitating the study of these components in flies.

PRC1 and PRC2 coregulate a variety of cellular processes including embryonic development, differentiation, and cell proliferation (Chan and Morey 2019; Loubiere et al. 2019, Loubiere et al. 2020). Consistent with a role for PcG proteins in cell identity, dysregulation of these components has been associated with multiple types of cancer (Piunti and Shilatifard 2021), including breast and prostate cancers, as well as hematologic malignancies (Varambally et al. 2002; Guo et al. 2007; Li et al. 2010; Zhang et al. 2010; Ntziachristos et al. 2012; Herviou et al. 2016; Kim and Roberts 2016; Althobiti et al. 2020). In agreement, PRC1 loss-of-function mutations in *Drosophila* result in up-regulation of major oncogenes including JAK/STAT, NOTCH, and JNK signaling pathways, which are important drivers of the tumorigenic process (Classen et al. 2009; Martinez et al. 2009; Loubiere et al. 2016, Torres et al. 2018).

Recent studies also suggest a role for PRC1 in DNA double-strand break (DSB) repair by homologous recombination (HR) and, to a lesser extent, non-homologous end joining (NHEJ) (Vissers et al. 2012). Upon exposure to ionizing radiation (IR) or FokI-induced DSBs, PRC1 core subunits are quickly and transiently recruited to the damage site in an ATM-dependent manner, where they induce H2A/H2AXK119ub and transcriptional silencing (Kakarougkas et al. 2014, 2015; Ui et al. 2015). This histone modification also promotes the recruitment of DSB repair components including 53BP1, BRCA1, RAP80, and the resection protein CtIP (Ismail et al. 2010, Pan et al. 2011, Ismail et al. 2013, Kakarougkas et al. 2014, Fitieh et al. 2021, Fitieh et al. 2022). However, the extent to which DSB repair relies on PRC1 remains unclear, as loss of the PRC1 subunit BMI-1 only partially affects repair kinetics and the resulting sensitivity to IR exposure is modest (Ismail et al. 2010, Fitieh et al. 2021).

Importantly, even a transient depletion of PRC1 core complex subunits leads to cancer formation in *Drosophila* (Parreno et al. 2024). Specifically, a 24-h depletion of the PRC1 subunit PH results in irreversible activation of key members of the JAK–STAT pathway, which in turn trigger a switch to a self-sustaining cancer cell fate, even upon restoration of normal PRC1 activity (Parreno et al. 2024). These EICs are proficient in DSB repair and do not show chromosome rearrangements or major increase in the mutational load (Parreno et al. 2024).

Here, we investigate whether a sustained inactivation of PRC1, which mimics a cancer-inducing context, eventually results in DNA damage repair defects and genomic instability. We show that inactivation of PH over 5 days is sufficient to induce massive over-replication, the misregulation of several repair genes, and a broad reduction in H2AK118ub and H3K27me3. Consistently, these tumors have elevated levels of endogenous DNA damage, DSB repair defects, and genomic instability. Together, these results are consistent with a model where EICs derived from transient PcG inactivation can rapidly transition to a state characterized by a highly genetically unstable genome. This instability might further contribute to tumor development when Polycomb depletion is maintained.

## Materials and methods

### *Drosophila* strains, genetics, and growth conditions

*Drosophila* flies were maintained on a standard corn-meal yeast extract medium at 25 °C. Crosses were performed as described in Parreno et al. (2024) (See also Fig. 1). Briefly, Gal80ts was used to achieve complete depletion of PH or the control *white* gene by switching the temperature from 18 °C to 29 °C. The *ey*-FLP system was used to generate complete knockdowns in the larval eye-antennal imaginal discs (EDs) (Parreno et al. 2024). Flies were reared and crossed at 18 °C to inhibit Gal4 activity. Six independent crosses were set up using 80 virgin females with 20 males for each genotype and egg laying was carried out for 4 h at 18 °C to synchronize the embryonic and larval development. As the timing of *Drosophila* development is temperature-dependent, we adapted the timing for each knockdown (KD) condition to carry out phenotypic and molecular analyses at comparable developmental times. Most dissections were performed on female larvae at the third instar larval stage (L3). Male larvae were used for the experiment described in Supplementary Fig. 2c,d. For achieving constant *ph*-KD and the temperature-matched *white*-KD control, tubes containing eggs were shifted to 29 °C throughout development, and third instar larvae (L3) were dissected 5 days after egg laying (AEL). The control no *ph*-KD was maintained at 18 °C throughout development (AEL to L3), with dissections typically done on day 11 AEL. For transient *ph*-KD at the L1 stage, flies were kept at 18 °C for 48 h, then shifted to 29 °C for 24 h and returned to 18 °C until dissection 11 days AEL (Parreno et al. 2024). For EdU experiments, transient *ph*-KD was induced at mid-L3 as follows: tubes were kept at 18 °C until 140 h AEL, shifted to 29 °C for 24 h and returned to 18 °C until dissection 24 h after the end of the temperature shift. Fly genotypes used  for *white*-KD control were: *ey-FLP, Act-gal4 (FRT.CD2 STOP)* (BL#64,095), *TubGal80ts* (BL#7019), and *UAS-wRNAi* (BL#33,623)/*UAS-GFP* (BL#64,095). Fly genotypes used for *ph*-KD were: *ey-FLP, Act-gal4 (FRT.CD2 STOP)* (BL#64,095), *TubGal80ts* (BL#7019), and *UAS-phRNAi* (VDRC#50,028)/*UAS-GFP* (BL#64,095).

### Immunostaining and fluorescence microscopy

Third instar female larvae were dissected to isolate eye-antennal imaginal discs (EDs) at room temperature (RT) in 1× PBS. Tissues were fixed in 4% formaldehyde for 30 min on a rotating wheel. Permeabilization was carried out for 1 h in 1× PBS containing 0.5% Triton X-100 on a rotating wheel. Blocking was performed for 1 h using 3% BSA PBST (1× PBS + 0.1% Triton X-100). Next, tissues were incubated with anti-γH2Av (1:500 prepared in 1% BSA PBST, Rockland, 600–401-914) for 2 h at RT. Samples were washed in 1× PBST for 15 min each for three times before adding a secondary antibody (donkey anti-rabbit Alexa Fluor 488, 1:1000 in 1% BSA PBST, Invitrogen, A-21206) for 2 h at RT, on a rotating wheel. Tissues were then washed in PBST for 15 min each for three times prior to DAPI staining at a final concentration of 1 µg/mL for 15 min. Discs were briefly washed in PBST and in 1× PBS for 5 min each. Discs were mounted in Vectashield medium (Eurobio scientific, catalog no. H-1000–10) or ProLong Gold antifade agent (Life Technologies, P36930). Images for quantification of DSB foci were taken with a DeltaVision deconvolution microscope (GE Healthcare/Leica) using a 60× oil immersion objective (Olympus PlanApo N, NA 1.42) and a CoolSNAP HQ2 camera. Images were deconvolved using SoftWoRx 6.0.

### EdU labeling to assess replication

Ethynyl-2′-deoxyuridine (EdU, thymidine analog) labeling was performed using Click-iT Plus EdU Alexa fluor 555 Imaging kit (Invitrogen, #C10638) as per manufacturer’s instructions. The EDs/tumors of female third instar larvae were dissected in Schneider's medium and EdU was added at a final concentration of 25 µM on a rotating wheel at RT for 15 min. After washing with PBS, tissues were fixed in 4% formaldehyde for 30 min and washed three times with PBS. The imaginal discs were permeabilized for 1 h in 1× PBS + 0.5% Triton X-100 on a rotating wheel then blocked for 1 h in 1× PBS + 0.1% Triton X-100 + 3% BSA. EdU detection was performed according to manufacturer’s instructions for 30 min on a rotating wheel at RT away from light. 500 µl of Click-iT reaction solution was prepared per tube containing 10–12 EDs/tumor. After a wash with 1× PBS + 0.1% Triton, DAPI staining was performed at a final concentration of 1 µg/ml for 15 min. Tissues were washes in 1× PBS + 0.1% Triton and discs were mounted in Vectashield medium. Image acquisition was performed using a Leica SP8-UV confocal microscope with a 10 × objective (NA 0.4) and 63× oil immersion objective [numerical aperture (NA 1.4)]. Quantification of EdU-positive cells in Supplementary Fig. 2b was done over a single plane of cells using Fiji.

### *Fluorescent *In Situ* Hybridization (FISH) for karyotype analysis*

Chromosome preparation and FISH was performed as previously described (Gatti and Goldberg 1991; Larracuente and Ferree 2015; Ryu et al. 2015). Briefly, EDs or tumors from L3 larvae were dissected in 0.7% NaCl solution and incubated in colchicine solution (3 ml of 0.7% NaCl + 100 µl of 1 mM colchicine) for 1 h at RT away from light. Following colchicine treatment, tissues were incubated in 0.5% NaOAc for 7 min, and fixed using freshly prepared 2.5% PFA in 45% acetic acid for 4 min on a coverslip. Tissues were squashed onto poly-lysine coated slides and snap frozen in liquid nitrogen. The slides were washed in 100% ethanol for 5 min, air dried, and stained with FISH probes for AACAC, AATAT, and 359-bp repeats as previously described (Larracuente and Ferree 2015). Probe sequences are: 5′-6-FAM-(AACAC)7, 5′-Cy3-TTTTCCAAATTTCGGTCATCAAATAATCAT, and 5′-Cy5-(AATAT)6. Imaging was performed with a DeltaVision deconvolution microscope (GE Healthcare/Leica) using a 60× oil immersion objective (Olympus PlanApo, NA 1.42) and a CoolSNAP HQ2 camera. Images were processed deconvolved using SoftWoRx 6.0.

### Ionizing radiation exposure to induce DNA damage

Early L3 female larvae were transferred into a petri dish containing standard food medium and were irradiated with the dose of 5 Gy of X-rays using a Precision X-RAD iR160 irradiator. After irradiation, larvae were maintained in the petri dish at 29 °C. Larval heads were dissected at indicated timepoints at RT in 1× PBS and fixed in 4% paraformaldehyde for 30 min before immunostaining. Microscopy and image analysis were performed as described above. Due to accelerated pupation of L3 stage larvae at 29 °C, DSB repair analysis was limited to 4 h post-irradiation.

### Bioinformatic analyses

All in-house bioinformatic analyses were performed using R version 3.6.3 (URL: https://www.R-project.org/) and are publicly available at https://github.com/vloubiere/Rawal_et_al_HCB_2024.git. Computations on genomic coordinate files and downstream analyses were conducted using the data.table R package (data.table: Extension of ‘data.frame’. https://r-datatable.com, https://Rdatatable.gitlab.io/data.table, https://github.com/Rdatatable/data.table, v1.14.2).

### Chromatin immunoprecipitation sequencing (ChIP-seq) and CUT&RUN data analysis

ChIP-seq datasets and the processed data files were downloaded from Gene Expression Omnibus (GEO) [GSE222193, (Parreno et al. 2024)], and are listed Supplementary Table 1. PH ChIP-seq, and H2AK118Ub and H3K27me3 CUT&RUN coverage was computed using 2.5 kb bins covering all canonical chromosomes (X, 2L, 2R, 3L, 3R, 4), and were visualized using Hilbert curves (Anders 2009) and an iteration level of 10. To compute enrichment ratios around the TSS of PcG-bound genes (−25 kb to + 75 Kb), H2AK118ub and H3K27me3 coverage was normalized to a set of activity-matched, unbound genes (*n* = 610 for each group).

### RNA-seq data analysis

RNA-seq datasets and the processed output files were obtained from GEO (GSE222193, (Parreno et al. 2024)), and are listed in Supplementary Table 1. Differential expression analysis output, performed using the DESeq2 R package (Love et al. 2014) (v1.26.0), was obtained from Parreno et al. (2024).

### GO terms enrichment

Gene Ontology (GO) terms associated to genes that were upregulated (*p*_adj_ < 0.05 and log_2_ fold change > 1) or downregulated (*p*_adj_< 0.05 and log_2_ fold change > 1) after constant or transient *ph*-KD were retrieved using the AnnnotationDbi R package (https://bioconductor.org/packages/AnnotationDbi.html, v1.48.0). For each GO term, over-representation was then assessed over a background set of genes consisting of all the genes that passed DESeq2 initial filters, using a one-sided Fisher’s exact test (alternative = “greater”). Obtained *p* values were corrected for multiple testing using false discovery rate (FDR). Differentially expressed genes associated to “cellular response to DNA damage,” “DNA repair,” and “DNA replication” GO terms are available in Supplementary Table 2, together with six other genes which were associated to the “cellular response to DNA damage stimulus,” which were nevertheless excluded from Fig. 3d due to the likelihood that their role in DNA damage response is indirect (Supplementary Table 2).

## Results

**Fig. 1:**
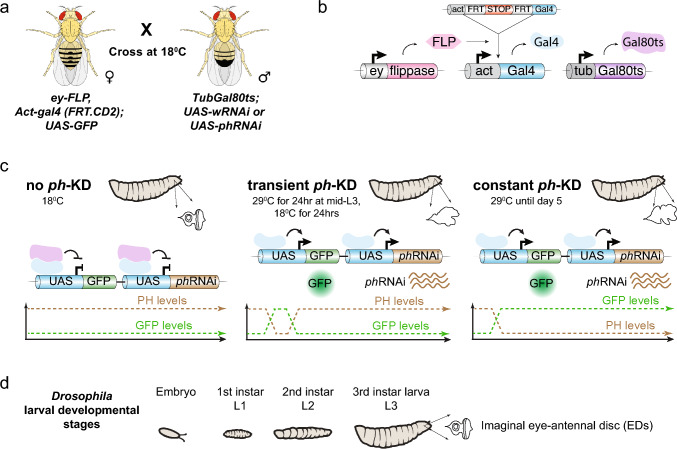
**Schematic representation of the experimental setup used to induce PRC1-dependent cancers.** **a** Scheme of the experimental cross used to generate progeny with thermosensitive conditional knockdown using *phRNAi* or *whiteRNAi*. Female virgins from the fly line with *ey-FLP*, *Act-gal4*, and *UAS-GFP* were crossed with males from the fly line with tub-Gal80ts and *UAS-phRNAi* or *UAS-wRNAi* at 18 °C. **b** Expression of the flippase *ey*-FLP (pink) in imaginal eye-antennal disc (ED) cells catalyzes the FLP out of a transcriptional stop (red) in the developing discs, allowing the expression of *act*-Gal4 (light blue). Constitutively expressed *tub*-Gal80ts (purple) encodes a temperature-sensitive Gal4 repressor. **c** At 18 °C, TubGal80ts inhibits Gal4-mediated *phRNAi* as well as *GFP* expression (used as internal control), thereby maintaining high levels of PH, leading to normal ED development. **d** Shift of the developing embryo/larvae to the restrictive temperature of 29 °C for 24 h or 5 days leads to transient or constant *ph*-KD, respectively, thereby inducing tumors, which can be dissected at the third instar stage of larval development (L3). Image prepared in Adobe Illustrator and Photoshop. Fly schemes are from Wikimedia Commons

### A fly system enables fine regulation of PH depletion during larval development

Recent studies showed that knocking down the PRC1 subunit PH for a short time (24 h, Fig. 1, transient *ph*-KD) during L1 larval stage is sufficient to induce EIC formation in third instar larvae (L3), and these EICs do not exhibit DNA repair defects or genomic instability (Parreno et al. 2024). These studies used an efficient thermosensitive *ph*-RNAi fly system to acutely deplete PH with a 24 h incubation time at 29 °C, and normal PH levels were restored within 48 h after switching to 18 °C (Parreno et al. 2024). We applied the same system to address the effect of prolonged PRC1 inactivation (constant *ph*-KD), thus enabling direct comparisons with transient *ph*-KD conditions. Constant *ph*-KD was obtained by incubating the larvae at 29 °C during the whole larval development for 5 days. Additionally, inactivation of the eye color-associated *white* gene (*white*-KD) or larvae maintained at 18 °C (no *ph*-KD) were used as controls (Fig. 1). Similar to transient *ph*-KD (Parreno et al. 2024), prolonged *ph*-KD also results in tumor formation in 100% of eye-antennal imaginal discs (EDs) of L3 larvae (Parreno et al. 2024).

### Prolonged *ph*-KD results in H2AK118ub and H3K27me3 loss at Polycomb target sites

**Fig. 2:**
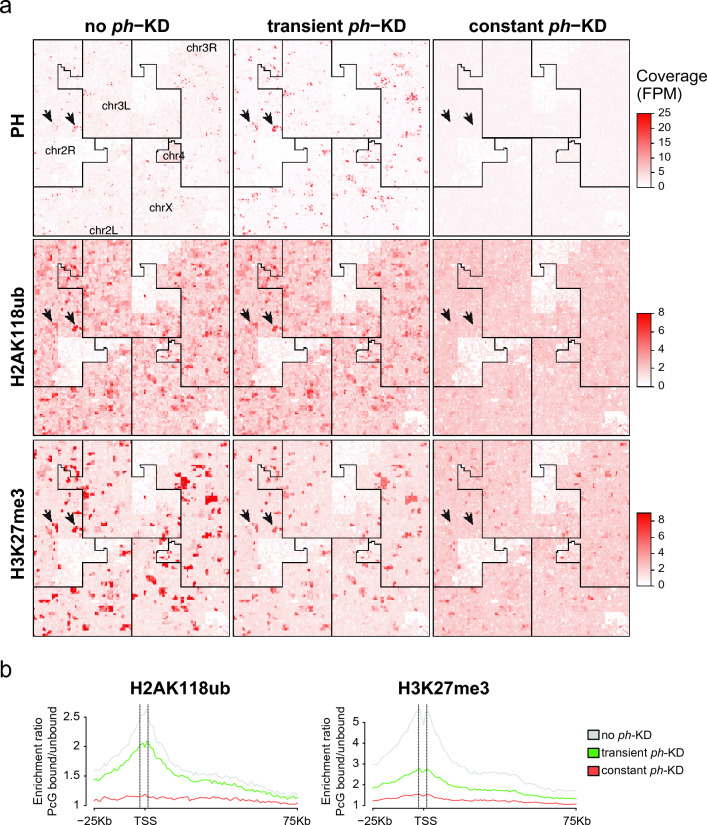
**Tumors induced by constant *ph*-KD display loss of H2AK118ub and H3K27me3 at PcG target genes.** **a** Hilbert curves display the enrichment of PH, H2AK118ub and H3K27me3 in control (no *ph*-KD), transient *ph*-KD, and constant *ph*-KD conditions, along all the chromosomes (see also Supplementary Fig. 1a). Black arrows indicate examples of enrichments of PH, H2AK118ub, and H3K27me3 at representative PRC1-target sites. Each chromosome/arm is outlined with a black line. Scales on the right indicate enrichment levels. FPM: fragments per million reads. **b** Enrichment ratios of H2AK118ub (left) and H3K27me3 (right) marks around the TSS (−25 to +75 Kb) of PcG-bound genes compared relative to a control set of activity-matched, PcG-unbound genes (*n* = 610 genes for each group) in tissues from indicated KD conditions

Given that both transient and constant *ph*-KD results in tumors characterized by loss of polarity and differentiation, we asked whether these tumors differ at the epigenetic level. We plotted the genome-wide enrichments of PH, H2AK118ub, and H3K27me3 from control EDs (no *ph*-KD), EICs after transient *ph*-KD, and tumors derived from constant *ph*-KD, using published ChIP-seq and CUT&RUN data sets [GSE222193 (Parreno et al. 2024), Supplementary Table 1]. Hilbert curves show that PH recruitment to chromatin is restored after transient *ph*-KD, whereas it is severely perturbed after constant *ph*-KD (Fig. 2a and Supplementary Fig. 1a). Consistently, the analysis of H2AK118ub and H3K27me3 enrichments around PRC1 target genes (PRC1-bound) relative to PRC1 non-target genes (PRC1-unbound) shows that these modifications are largely restored after transient *ph*-KD, but not after constant *ph*-KD (Fig. 2b and Supplementary Fig. 1b). The most significant difference between EICs derived from transient *ph*-KD and constant *ph*-KD tumors is associated with H2AK118ub, consistent with this histone modification being the primary modification established by PRC1 (Fig. 2a,b and Supplementary Fig. 1b). We conclude that tumors resulting from prolonged *ph*-KD are characterized by extensive loss of H2AK118ub and H3K27me3 at PcG target genes, while this is not the case for EICs resulting from transient *ph*-KD.

### Prolonged *ph*-KD results in upregulation of DNA replication and repair genes

**Fig. 3:**
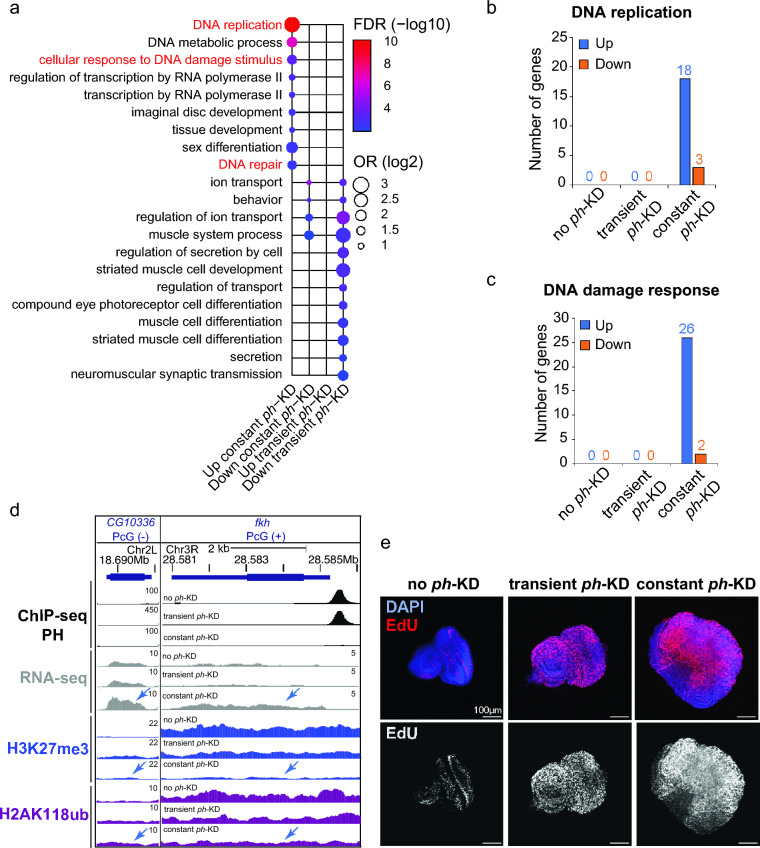
**Tumors induced by constant** ***ph*-KD display dysregulation of DNA damage response- and replication -associated genes.** **a** Representative GO terms enriched in genes differentially expressed after constant *ph*-KD or transient *ph*-KD (up- or down- regulation). The complete list is shown in Supplementary Fig. 1c. **b** Number of genes specifically dysregulated after constant *ph*-KD associated with “DNA replication” GO term. **c** Number of genes specifically dysregulated after constant *ph*-KD and associated with “DNA damage response” GO term. **d** Genome browser snapshots for representative genes upregulated only in constant *ph*-KD conditions showing PH, H2AK118ub, and H3K27me3 normalized tracks (ChIP-seq or CUT&RUN, normalized by input) and gene expression by RNA-seq, in control (no *ph*-KD), transient *ph*-KD, and constant *ph*-KD conditions. **e** EdU staining of EDs and tumors from L3 in indicated KD conditions

Given the major epigenetic differences between EICs generated by transient and constant *ph*-KD, we examined the differential gene expression between these tumors compared to control tissues (no *ph*-KD) and temperature-matched *white*-KD, using the published datasets derived from RNA-seq analyses (Parreno et al. 2024). As shown in Fig. 3a and Supplementary Fig. 1b, we found significant differences in gene expression profiles between transient and constant *ph*-KD tumors. These include the upregulation of genes required for tissue and organ development in constant *ph*-KD tumors relative to transient *ph*-KD tumors, consistent with PRC1 roles in organismal development (Loubiere et al. 2019, Loubiere et al. 2020).

Remarkably, gene clusters corresponding to Gene Ontology (GO) terms related to DNA replication, DNA damage, and DNA repair were also mostly upregulated in constant *ph*-KD conditions relative to transient *ph*-KD tumors (Fig. 3a). Consistently, a fold-change analysis of all the genes classified as “DNA replication” (*n* = 111) or “DNA damage response” (*n* = 242) shows a significantly higher level of transcription for both categories in constant *ph*-KD tumors relative to control, and also compared to all genes (Supplementary Fig. 1c). This indicates that DNA replication and DNA damage response genes are overall more transcriptionally active in tumors derived from sustained *ph*-KD.

Within this general trend, 21 genes required for “DNA replication” and 28 genes required for the “DNA damage response” were the most affected, displaying at least a twofold change in expression in constant *ph*-KD tumors relative to controls, most of which (18 and 26 genes, respectively) were upregulated (Fig. 3b, c).

Most of the DNA replication and DNA damage response genes upregulated in tumors derived from constant *ph*-KD are not associated with PRC1 enrichments in normal tissues (no *ph*-KD) (e.g., Fig. 3d, CG10336 or TIPIN in mammals), suggesting that they are not direct targets of PH and their upregulation is an indirect effect of PRC1 loss. The most notable exception is the gene for the replication, repair, and transcription factor Fkh (FOXA2 and FOXA1 in mammals) (Knott et al. 2012, Li et al. 2012, Dummer et al. 2016, Jin et al. 2020, Hoggard et al. 2021), whose promoter is enriched for PRC1 in normal tissues. This suggests that PRC1 downregulation from constant *ph*-KD directly affects *fkh* expression (Fig. 3d).

The replication genes affected in constant *ph*-KD tumors correspond to key replication components, including the MCM complex, origin firing factors, and several DNA polymerases (Supplementary Table 2). This increase in expression of replication-linked genes might result from an overall induction of replication in the tissue. Thus, we investigated the proliferation state of the cells in these tumors by EdU staining. As expected, control EDs are characterized by a few replicating cells posteriorly to the morphogenetic furrow (Avellino et al. 2023; Parreno et al. 2024) (Fig. 3a and Supplementary Fig. 2a,b). Conversely, tumors derived from constant *ph*-KD are characterized by massive EdU incorporation, indicating the switch to an uncontrolled over-proliferating state (Fig. 3a and Supplementary Fig. 2a,b). Of note, DNA replication-associated genes are found over-expressed also in transient *ph*-KD tumors (Supplementary Fig. 1d), albeit to a lesser extent compared to constant *ph*-KD tumors. Consistently, transient *ph*-KD tumors are also enriched for replicating cells compared to controls (Parreno et al. 2024) (Fig. 3e and Supplementary Fig. 2a,b), although the number of replicating cells in these tumors is lower compared with tumors derived from constant *ph*-KD (Supplementary Fig. 2a,b).

Together, these results establish that constant *ph*-KD leads to tumors characterized by the upregulation of several DNA replication genes, which is likely a consequence of cell hyperproliferation. This upregulation is more pronounced than that observed in tumors derived from transient *ph*-KD, consistent with an higher proliferation rate. Upregulation of components required for replication initiation and progression can also contribute to the acquisition of the hyperproliferative state (Yu et al. 2020). In addition, we observed dysregulation of several DNA damage response genes upon constant depletion of PH, most of which are likely the indirect consequence of PH loss. These genes are mostly expressed at normal levels in transient *ph*-KD tumors, representing a major difference between the effects of short-term and long-term PH depletions.

### Prolonged *ph*-KD leads to defective DSB repair and increased genomic instability

DNA repair genes over-expressed in constant *ph*-KD tumors include several components previously linked to damage accumulation, cancer formation, and/or poor cancer prognosis (Table 1),

**Table 1:** **List of DNA repair genes dysregulated genes upon constant *ph*-KD.** Selected genes from Supplementary Table 2, including their DNA repair function, mammalian homologs, and link to cancer. Mismatch repair (MMR), base excision repair (BER), nucleotide excision repair (NER), translesion synthesis (TLS)

*Drosophila *gene	Mammalian homolog	Function in DNA repair	Link to cancer
*Upregulated*			
Mms4	EME1	Holliday Junction resolvase in complex with Mus81. Replication fork processing and repair	Overexpressed in several cancers, including colorectal and lung cancer. Associated with poor prognosis and chemo resistance
RecQ4	RECQ4	HR repair	Overexpressed in several cancers, including osteosarcomas, prostate, colorectal, and breast cancers. Associated with poor prognosis and chemo resistance
PolH	POLH	Translesion polymerase	Overexpressed in several cancers, including breast, lung, ovarian and bladder cancers. Associated with poor prognosis and chemoresistance
CG43295	MRNIP	HR repair through phase separation, fork protection	Overexpressed in colorectal cancer, associated with radioresistance and poor prognosis
FANCI	FANCI	Interstrand crosslink repair, stalled fork processing	Overexpressed in several cancers, including lung adenocarcinoma, cervical cancer and liver hepatocellular carcinoma, associated with poor cancer prognosis
Spel1 Mlh1 Msh6	MSH2 MLH1 MSH6	Mismatch repair, Homeologous recombination	Commonly overexpressed in cancer, correlated with poor prognosis in prostate cancer
Rif1	RIF1	Telomere maintenance, DSB repair (prevents resection, promoting NHEJ)	Commonly overexpressed in cancer, promotes drug resistance, correlated with poor prognosis
CG10336 Timeout/tim2	TIPIN TIMELESS	Fork protection complex, Replication stress response, Homologous recombination repair, Telomere maintenance	Overexpression induces cancer, promotes drug resistance, associated with poor cancer prognosis
Claspin	CLASPIN	Checkpoint activation in response to replication stress	Overexpression induces cancer, promotes radioresistance, associated with poor prognosis
*Downregulated*			
PCNA2	PCNA	Sliding clamp for DNA repair in *Drosophila*. HR, MMR, NER, BER, TLS,	Downregulated in many cancers, particularly in sarcomas and testicular cancer

like Mms4 (Dewalt et al. 2014), RecQ4 (Maire et al. 2009; Su et al. 2010; Xu et al. 2021), PolH (Tomicic et al. 2014, Sonobe et al. 2024), Tipin/Timeless (Zhou et al. 2020; Chen et al. 2022), Claspin (Choi et al. 2014), MRNIP (Staples et al. 2016, Bennett et al. 2020, Wang et al. 2022), FANCI (Smogorzewska et al. 2007; Li et al. 2023), MMR proteins (Msh2, Mlh1, Msh6) (Shcherbakova and Kunkel 1999; Velasco et al. 2002; Li et al. 2008; Wagner et al. 2016; Wilczak et al. 2017; Chakraborty et al. 2018; Donis et al. 2021; Zhou et al. 2024), and Rif1 (Liu et al. 2018; Mei et al. 2018; Sad et al. 2021). Similarly, genes downregulated in constant *ph*-KD tumors include known components required for DNA repair and replication fork protection in the presence of replication damage, such as the PCNA variant PCNA2 (Feng et al. 2023) (Table 1). Collectively, misregulation of these genes is expected to lower fork protection, increase DSB formation in response to stalled fork, and impair DSB repair.

**Fig. 4:**
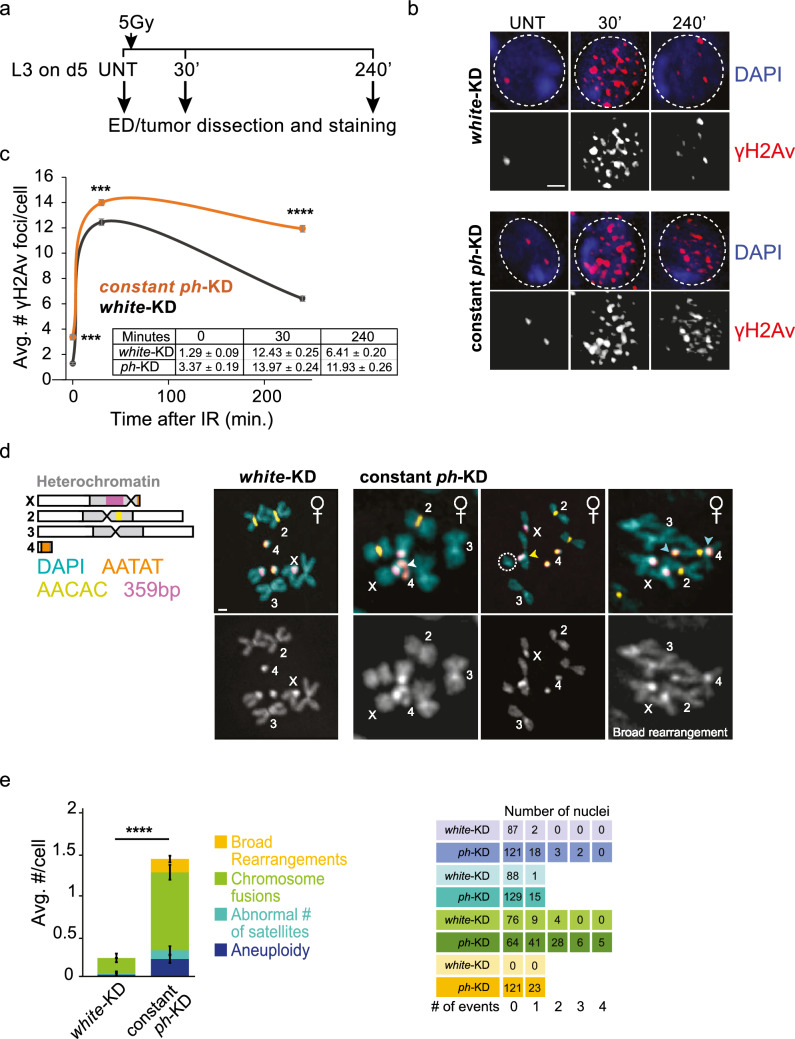
**Constant *ph*-KD tumors are characterized by DSB repair defects and genomic instability.** **a** Schematic representation of the experiment used to assess DSB repair. **b** Representative images of *Drosophila* cells from EDs or tumors, stained for γH2Av before (UNT) and at the indicated timepoints after IR, from *white*-KD (control) and constant *ph*-KD conditions. Dashed circles indicate the position of each nucleus, identified by DAPI staining. **c** Quantification of the number of γH2Av foci per cell before (0 min) and after irradiation (30 and 240 min) in EDs or tumors derived from *white*-KD (control) and constant *ph*-KD. *n* ≥ 100 cells per replicate, representing EDs or tumors from three distinct larvae and independent crosses, at the indicated time points from IR exposure. Error bars, standard error of the mean (SEM). Statistical significance was calculated using a two-sided *t*-test: ****p value < 1 × 10^−5^. The table show the individual average values and corresponding SEM. **d** Examples of karyotypes from *white*-KD (control) and constant *ph*-KD EDs or tumors from female larvae, showing examples of different chromosomal abnormalities. The scheme of the chromosomes shows the position of the major satellites stained by FISH. White arrowheads: fusions between Chr 4 and Chr X, and between two Chr 4. Dashed circle: chromosome fragment derived from a fusion between Chr 3 and X (yellow arrowhead). Cyan arrowheads: fusions between Chr 4 and Chr 2, in addition to broad rearrangements. **e** Quantification of chromosome abnormalities in EDs or tumors from *white*-KD (control) and constant *ph*-KD flies. For each type of abnormality (see color legend), the number of counted events are shown on the right. *n* = 89 karyotypes representing EDs from three larvae from independent crosses for *white*-KD. *n* = 144 karyotypes representing tumors from seven larvae from independent crosses for *ph*-KD. Error bars, SEM. ****p value < 1 × 10^−5^. Statistical significance was calculated using a two-sided *t*-test. Scale bars ,1 µm

We directly tested this by investigating DNA break formation through immunofluorescence (IF) analysis of γH2Av foci in tumors dissected from L3 larvae after constant *ph*-KD or in EDs from the temperature-matched *wRNAi* control. Constant *ph*-KD results in a threefold increase in the number of γH2Av foci in the tissue, indicating a higher level of endogenous DNA damage (Fig. 4a–c). This likely derives from the higher number of replicating cells, which typically experience a higher baseline level of damage than non-replicating cells, along with defective fork protection and repair.

In addition, we investigated the DSB repair response by treating constant *ph*-KD tumors and their controls with 5 Gy ionizing radiation (IR), and by quantifying the kinetics of γH2Av focus formation and resolution (Fig. 4c). Both tumor and ED control tissues showed a significant increase in the number of γH2Av foci 30 min after IR, indicating DSB induction and checkpoint activation. The higher level of repair foci in *ph*-KD tumors relative to the control reflects the higher baseline level of damage (Fig. 4c, timepoint 0). Importantly, constant *ph*-KD tumors display a significantly higher number of γH2Av foci relative to control EDs 4 h after irradiation, and this difference is much more pronounced than what is observed in untreated (UNT, timepoints 0) tissues or in tissues fixed 30 min after IR (Fig. 4b,c). This indicates that, unlike transient *ph*-KD tumors (Parreno et al. 2024), constant *ph*-KD tumors are defective in DSB repair.

Given the higher amount of DNA damage and defective repair, we hypothesized that constant *ph*-KD tumors might accumulate unrepaired DSBs over time, resulting in chromosome rearrangements and genomic instability. We tested this by karyotype analysis of tumors from constant *ph*-KD and EDs from *wRNAi* control in L3 larvae (Fig. 4d). We stained with FISH probes for pericentromeric regions of different chromosomes to facilitate chromosome detection in rearranged conditions. Remarkably, we observe a six-fold increase in the frequencies of chromosome rearrangements in constant *ph*-KD tumors relative to controls (Fig. 4d,e). Rearrangements include a large number of chromosome fusions, aneuploidies, and abnormal number of satellites (Fig. 4d,e). Moreover, we observe a significant increase in a rare form of rearrangements characterized by fusions across several chromosomes (“broad rearrangements”) (Fig. 4d, e). The increase in chromosome rearrangements occurred in both males and females, suggesting that the effect is not sex-specific (Supplementary Fig. 2c,d).

In conclusion, tumors induced by PH depletion over 5 days during larval stages are characterized by misregulation of genes required for replication fork protection and DNA repair, DSB repair defects, and widespread genome instability, which was not observed in EICs derived from transient *ph*-KD.

## Discussion

**Fig. 5:**
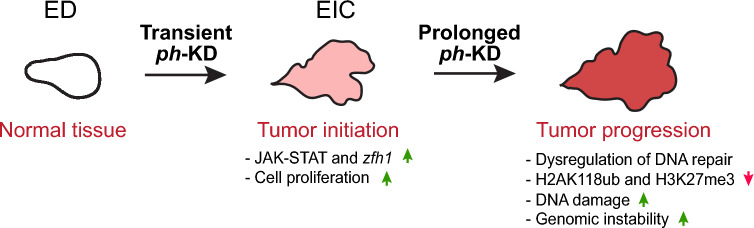
**Model for tumor progression in EICs.** Upon transient ph-KD, EDs switch to a hyperproliferative cell fate notably due to an irreversible activation of the JAK–STAT pathway and *zfh1* (Parreno et al. 2024). Prolonging *ph*-KD for 4 additional days results in accumulation of replication damage, misregulation of DNA damage response genes, defective DSB repair leading to persistent DNA damage. This progression reflects a cascade of events where initial hyperproliferation leads to increased replication stress and subsequent dysregulation of DNA repair mechanisms, culminating in genome instability

Chromosomal instability is a common hallmark of both human (Hanahan and Weinberg 2011) and fly tumors (Gateff and Schneiderman 1974; Basto et al. 2008; Torres et al. 2010; Dekanty et al. 2012), and it can contribute to tumor invasiveness (Barrio et al. 2023). However, the cause–effect relationship between abnormal karyotypes and tumor progression remains debatable [reviewed in (Fukasawa 2005; Milan et al. 2014)] and consistently, how tumors associated with PRC1 dysregulation acquire genome instability was unclear. Our comparative analysis of epigenetically initiated cancers due to transient *ph*-KD and tumors resulting from prolonged *ph*-KD offers a rare opportunity to identify progressive changes occurring in a developing tumor. These studies shed light on how epigenetic tumors with a stable genome can quickly transition into a state characterized by massive genomic instability through prolonged PRC1 inactivation (Fig. 5).

We show that, unlike transient *ph*-KD (Parreno et al. 2024), constant *ph*-KD results in loss of H2AK118ub and H3K27me3 at Polycomb target genes, dysregulation of several DNA repair genes, marked defects in DSB repair, and widespread genome instability. Importantly, the transition to a tumor characterized by an unstable genome is reached within only 5 days of PH depletion, revealing a rapid acquisition of this typical cancerous phenotype.

However, transient *ph*-KD tumors already display a hyperproliferating state and some level of misregulation of replication genes. This suggests a progression of the tumor where the hyperproliferating state is acquired first, resulting in a higher baseline level of damage, followed by dysregulation of fork protection and repair genes (including PRC1 itself), which in turn results in DNA repair defects and chromosome rearrangements. Loss of PRC1 function can contribute to these phenotypes in non-mutually exclusive ways: (i) by increasing transcription globally, thus bolstering replication stress form replication–transcription collision (Zeman and Cimprich 2014, Hamperl et al. 2017, Gomez-Gonzalez and Aguilera 2019, Chakraborty et al. 2023); (ii) by preventing the establishment of H2AK118ub and H3K27me3 at DSBs, thus interfering with DSB repair (Ismail et al. 2010, Campbell et al. 2013, Ismail et al. 2013, Fitieh et al. 2021, Fitieh et al. 2022); and (iii) by misregulating the expression of genes required for replication, DNA fork protection and DSB repair, thus increasing the accumulation of unrepaired and misrepaired breaks. In addition, these defects are amplified in a context of a hyperproliferating tissue, with additional potential for replication damage. Collectively, tumors derived from transient or constant *ph*-KD represent a promising model system to investigate the gradual epigenetic and genomic changes leading to cancer formation.

Together, these observations also highlight the importance of core PRC1 subunits as tumor suppressors and guardians of genome stability. The finding that transient PRC1 depletion leads to epigenetic tumors without inducing genome instability, while prolonged inactivation of this complex results in DNA repair defects and massive genome rearrangements, is also important to inform cancer treatment approaches. PRC1 has been considered a potential therapeutic target for cancer (Shukla et al. 2021; Itoh et al. 2022; Park et al. 2023) and our study suggests that PRC1 inactivation will likely increase the sensitivity of tumor cells to DNA damaging agents. On the other hand, “epi-drugs” targeting PRC1 can also potentially transform healthy tissues into epigenetically initiated cancers and induce genome instability in response to protracted treatments. Thus, understanding how epigenetic tumors acquire a state characterized by high genome instability is important for establishing improved and safer approaches for cancer therapy.

### Supplementary Information

Below is the link to the electronic supplementary material.

Supplementary file 1 Fig. 1 **Constant *ph*-KD leads to reduced H2AK118ub and H3K27me3 at PcG targets genes and dysregulation of DNA damage response/replication genes.** **a** Scheme showing the relative position of fly chromosomes in the Hilbert curve, including an estimate of the position of uniquely mapped sequences corresponding to pericentromeric regions (gray). **b** Box plots showing the ratio of H2AK118b and H3K27me3 enrichments across PcG-bound genes relative to PcG-unbound genes (*n* = 610 genes) in control (no *ph*-KD), transient *ph*-KD, or constant *ph*-KD conditions. ****p < 1 × 10^−5^ by two-sided Wilcoxon text. Box plots depict the median (line), upper and lower quartiles (box) ±1.5× interquartile range (whiskers) and outliers are not shown. **c** Complete list of GO terms enriched in genes differentially expressed (up- or downregulated) after constant or transient *ph*-KD. **d** Violin plot highlighting the transcriptional fold change (log_2_) in control (no *ph*-KD), transient, and constant *ph*-KD conditions, for GO terms “DNA damage response” (*n* = 242) and “DNA replication” (*n* = 111), compared with genes representing all GO terms. n.s., not significant; ****p < 1 × 10^−5^ by one-sided Fisher’s exact test (alternative= greater, FDR correction for multiple testing). (EPS 2705 KB)

Supplementary file 2 Fig. 2 **Transient or constant *ph*-KD results in cell over-proliferation.** **a** Representative images of EdU staining of EDs or tumors from L3 for indicated genotypes and temperatures. Images are projections of a few Z-stacks. **b** Quantification of Edu-positive cells in indicated tissues. *n* = 100 cells per tissue from three independent tissues. Error bars, SEM. Statistical significance was calculated using a two-sided *t*-test: **p value < 1 × 10^−2^, ***p value < 1 × 10^−3^, ****p value < 1 × 10^−5^. Scale bars, 5 µm. **c** Karyotypes from white-KD (control) and constant *ph*-KD EDs per tumors of male larvae, showing examples of different genomic abnormalities. The scheme of the chromosomes shows the position of the major satellites stained by FISH. White arrowheads: fusions between Chr 4 and Chr 3 or Chr4 and Chr Y. Orange arrowheads and zoomed details: fusion between two Chr 4. Yellow arrowhead and zoomed detail: additional copies of AACAC repeats on Chr Y. This might also indicate a rearrangement between Chr Y and 2, given that a Chr 2 is missing in this Karyotype (aneuploidy). **d** Quantification of chromosome abnormalities in EDs or tumors from white-KD (control) and constant *ph*-KD male and female larvae. For females, *n* = 49 karyotypes representing EDs from one larva for white-KD. A total of *n* = 87 karyotypes representing tumors from five larvae from independent crosses for *ph*-KD. For males, *n* = 40 karyotypes representing EDs from two larvae from independent crosses for white-KD. A total of *n* = 57 karyotypes representing tumors from two larvae from independent crosses for *ph*-KD. Error bars, SEM. ****p value < 1 × 10^−5^. Statistical significance was calculated using a two-sided *t*-test. Scale bars, 1 µm. (EPS 17710 KB)

Supplementary file 3 (DOCX 17 KB)

Supplementary file 4 Table 2 Complete list of differentially expressed genes representing the GO terms “cellular response to DNA damage,” “DNA repair,” and “DNA replication.” The list include the log_2_ fold change after no *ph*-KD (control, referred as PH18), transient *ph*-KD (referred as PHD11), or constant *ph*-KD (referred as PH29), relative to temperature-matched controls. A manually curated list of differentially expressed genes is used for Fig. 3b,c. (XLSX 95 KB)

## Data Availability

Data from this study will be available from the corresponding authors upon reasonable request.

## Code availability

All custom scripts were made publicly available at https://github.com/vloubiere/Rawal_et_al_HCB_2024.git
