# Supplementary material for: Sustained inactivation of the Polycomb PRC1 complex induces DNA repair defects and genomic instability in epigenetic tumors
Source: Histochem Cell Biol. 2024 Jun 18;162(1-2):133–47. doi: 10.1007/s00418-024-02302-z (PMC11227471; doi:10.1007/s00418-024-02302-z)
Supplement: Supplementary file 3 — (DOCX 17 KB) [file 418_2024_2302_MOESM3_ESM.docx]

**Supplementary Table 1 List of sequencing datasets used in this study.**

| Accession number | Description | Figure |
| --- | --- | --- |
| [GSM6917477](https://www.ncbi.nlm.nih.gov/geo/query/acc.cgi?acc=GSM6917477)-79 | RNA, Control no ph-KD, rep1-3 | Fig. 3, Supp Fig. 1 |
| [GSM6917480](https://www.ncbi.nlm.nih.gov/geo/query/acc.cgi?acc=GSM6917480)-82 | RNA, Constant ph-KD, rep1-3 | Fig. 3, Supp Fig. 1 |
| [GSM6917483](https://www.ncbi.nlm.nih.gov/geo/query/acc.cgi?acc=GSM6917483)-85 | RNA, Transient ph-KD D11 AEL, rep1 | Fig. 3, Supp Fig. 1 |
| [GSM6917468](https://www.ncbi.nlm.nih.gov/geo/query/acc.cgi?acc=GSM6917468)-70 | RNA, Control no white-KD constGFP, rep1-3 | Fig. 3, Supp Fig. 1 |
| [GSM6917471](https://www.ncbi.nlm.nih.gov/geo/query/acc.cgi?acc=GSM6917471)-73 | RNA, Constant white-KD constGFP, rep1-3 | Fig. 3, Supp Fig. 1 |
| [GSM6917474](https://www.ncbi.nlm.nih.gov/geo/query/acc.cgi?acc=GSM6917474)-76 | RNA, Transient white-KD constGFP, rep1-3 | Fig. 3, Supp Fig. 1 |
| [GSM6917498](https://www.ncbi.nlm.nih.gov/geo/query/acc.cgi?acc=GSM6917498)-99 | CUT&RUN H2AK118Ub, Control no ph-KD, rep1-2 | Fig. 2, Supp Fig. 1 |
| [GSM6917500](https://www.ncbi.nlm.nih.gov/geo/query/acc.cgi?acc=GSM6917500)-01 | CUT&RUN H2AK118Ub, Constant ph-KD, rep1-2 | Fig. 2, Supp Fig. 1 |
| [GSM6917502](https://www.ncbi.nlm.nih.gov/geo/query/acc.cgi?acc=GSM6917502)-03 | CUT&RUN H2AK118Ub, Transient ph-KD D11 AEL, rep1-2 | Fig. 2, Supp Fig. 1 |
| [GSM6917510](https://www.ncbi.nlm.nih.gov/geo/query/acc.cgi?acc=GSM6917510)-11 | CUT&RUN H3K27me3, Control no ph-KD, rep1-2 | Fig. 2, Supp Fig. 1 |
| [GSM6917512](https://www.ncbi.nlm.nih.gov/geo/query/acc.cgi?acc=GSM6917512)-13 | CUT&RUN H3K27me3, Constant ph-KD, rep1-2 | Fig. 2, Supp Fig. 1 |
| [GSM6917514](https://www.ncbi.nlm.nih.gov/geo/query/acc.cgi?acc=GSM6917514)-15 | CUT&RUN H3K27me3, Transient ph-KD D11 AEL, rep1-2 | Fig. 2, Supp Fig. 1 |
| [GSM6917516](https://www.ncbi.nlm.nih.gov/geo/query/acc.cgi?acc=GSM6917516)-21 | CUT&RUN IgG, Control no ph-KD, constant ph-KD and Transient ph-KD, rep 1-2 each | Fig. 2, Supp Fig. 1 |
| [GSM6917522](https://www.ncbi.nlm.nih.gov/geo/query/acc.cgi?acc=GSM6917522)-23 | ChIP PH, Control no ph-KD, rep1-2 | Fig. 2, Supp Fig. 1 |
| [GSM6917524](https://www.ncbi.nlm.nih.gov/geo/query/acc.cgi?acc=GSM6917524)-25 | ChIP PH, Constant ph-KD, rep1-2 | Fig. 2, Supp Fig. 1 |
| [GSM6917526](https://www.ncbi.nlm.nih.gov/geo/query/acc.cgi?acc=GSM6917526)-27 | ChIP PH, Transient ph-KD D11 AEL, rep1-2 | Fig. 2, Supp Fig. 1 |
| [GSM6917528](https://www.ncbi.nlm.nih.gov/geo/query/acc.cgi?acc=GSM6917528)-33 | ChIP INPUT, Control no ph-KD, constant ph-KD and Transient ph-KD, rep1-2 each | Fig. 2, Supp Fig. 1 |
